# Reducing heat load density with asymmetric and inclined double-crystal monochromators: principles and requirements revisited

**DOI:** 10.1107/S1600577524009755

**Published:** 2025-01-01

**Authors:** XianRong Huang, Lahsen Assoufid, Albert T. Macrander

**Affiliations:** a Advanced Photon Source Argonne National Laboratory (ANL) 9700 South Cass Avenue Lemont IL60439 USA; SLAC National Accelerator Laboratory, USA

**Keywords:** inclined double-crystal monochromator, asymmetric monochromator, dynamical theory, non-coplanar diffraction, high heat load

## Abstract

Asymmetric double-crystal monochromators (aDCMs) and inclined DCMs (iDCMs) can significantly expand the X-ray beam footprint and consequently reduce the heat load density and gradient. Based on rigorous dynamical theory calculations, the major principles and properties of aDCMs and iDCMs are presented to guide their design and development, particularly for fourth-generation synchrotrons. In addition to the large beam footprint, aDCMs have very large bandwidths (up to ∼10 eV) and angular acceptance, but the narrow angular acceptance of the second crystal requires precise control of the relative orientations and strains. Based on Fourier coupled-wave diffraction theory calculations, it is rigorously proved that the iDCM has almost the same properties as the conventional symmetric DCM, including the efficiency, angular acceptance, bandwidth, tuning energy range and sensitivity to misalignment. The exception is that, for the extremely inclined geometry that can achieve very large footprint expansion, the iDCM has (beneficially) a larger bandwidth and wider angular acceptance. Inclined diffraction has the ‘rho-kick effect’ that can be cancelled by the second reflection of the iDCM (even with misalignment), except that inhomogeneous strains may cause non-uniform rho-kick angles. At present, fabrication/mounting-induced strains pose low risk since they can be controlled to <0.5 µrad over large areas. The only uncertain challenge is the thermally induced strains, yet it is estimated that these strains are naturally lowered by the large footprint and may be further mitigated by optimized cryogenic cooling to the 1–2 µrad level. Overall, aDCMs and iDCMs have more stringent requirements than normal DCMs, but they are feasible schemes in practice.

## Abstract 2

The major principles and requirements of asymmetric and inclined double-crystal monochromators are re-examined and presented to guide their design and development for significantly reducing heat load density and gradient on the monochromators of fourth-generation synchrotron light sources and X-ray free-electron lasers.

## 1. Introduction

The emerging fourth-generation synchrotron light sources and X-ray free-electron lasers with increased brilliance are promising to significantly improve the performance of various X-ray diffraction, scattering and imaging techniques. Among the unique properties of these new light sources are the small electron beam sizes and emittance that can produce X-ray beams with small dimensions along both the vertical and horizontal directions (Tavares *et al.*, 2018 ▸; Raimondi *et al.*, 2023 ▸), *i.e.* the natural X-ray beam cross section becomes nearly a round dot and the beam footprint on the high-heat-load double-crystal monochromator (DCM) can be about 1 mm × 1 mm or smaller at the insertion device beamlines (APS-U, 2019 ▸). In comparison, the beam shape is generally an extended horizontal line for third-generation sources. Although the total power may not differ significantly from that of the third-generation one, the smaller beam size of the fourth-generation light source obviously produces much higher heat load density on the DCMs. Such highly localized heat load can induce severe heat bumps (Revesz *et al.*, 2007 ▸), which correspond to large lattice constant gradients and lattice plane tilts (usually at the µrad level or more even for third-generation synchrotrons) (Chumakov *et al.*, 2014 ▸) that are equivalent to the slope errors of mirrors and may significantly reduce the DCM efficiency (flux), broaden the virtual source (Huang *et al.*, 2012 ▸), and degrade the X-ray coherence and wavefront.

In addition to improving the cooling techniques of DCMs, an effective strategy for reducing the heat bump is to use small-incidence diffraction geometry to expand the X-ray beam footprint on the crystal for spreading the heat load. For this purpose, mainly two types of DCMs, asymmetric DCMs (aDCMs) and inclined DCMs (iDCMs), have been proposed in the literature (Kohra, 1962 ▸; Nave *et al.*, 1995 ▸; Khounsary, 1992 ▸; Hrdý, 1992 ▸; Macrander *et al.*, 1992 ▸; Macrander *et al.*, 1993 ▸). Regular DCMs and aDCMs in the vertical diffraction configuration magnify only the vertical beam size (beam height), but an iDCM magnifies both the beam height and the beam width (Khounsary, 1992 ▸; Macrander *et al.*, 1992 ▸). Thus, footprint magnification of the horizontal incident beam size (beam width) is unique to iDCMs and is particularly suited to the smaller beam widths of fourth-generation light sources. However, aDCMs and iDCMs have seldom been implemented (Bernstorff *et al.*, 1998 ▸; Yabashi *et al.*, 1999 ▸; Tajiri *et al.*, 2019 ▸). There are several reasons for this situation. For aDCMs, a limitation is that the asymmetrically cut surface reduces the energy tuning range. Another reason might be that, compared with symmetric DCMs, aDCMs may have special requirements that have been overlooked in the literature. By contrast, an iDCM has almost the same energy tuning range as the conventional symmetric DCM. However, theoretical calculations of the involved non-coplanar diffraction configuration (*i.e.* the incident wavevector, the diffraction vector and the crystal surface normal are not in the same plane) are extremely difficult using the conventional dynamical theory. Therefore, most of the previous work was on the geometrical diffraction properties of iDCMs (Kashihara *et al.*, 1998 ▸; Hrdý *et al.*, 1995 ▸). Macrander *et al.* (1993 ▸) have used advanced dynamical theory methods to compute the non-coplanar inclined diffraction case, but the computation was limited to a single bounce. To fully understand the practical requirements of an iDCM, modelling and accurate calculations of the two-bounce configuration are required to include possible mis­alignment between the two crystals along all the pitch, roll and yaw axes. The extremely inclined geometry with the inclined angle of the iDCM very close to 90° also needs accurate calculations as this configuration is critical for achieving very large footprint expansion (Khounsary, 1992 ▸; Macrander *et al.*, 1992 ▸). Without such rigorous calculations, the efficiency, the angular acceptance, the bandwidths and the mechanical requirements of iDCMs are unclear. Consequently, there have been many concerns or even misunderstandings about iDCMs.

In this paper, we revisit these two types of DCMs by providing systematic re-examinations of their detailed principles and requirements. We demonstrate that these two types of DCMs are, in fact, feasible and easy-to-implement schemes for efficiently reducing the heat load density of DCMs for fourth-generation synchrotrons, together with many other benefits.

## 2. Properties of aDCMs

**Figure 1:**
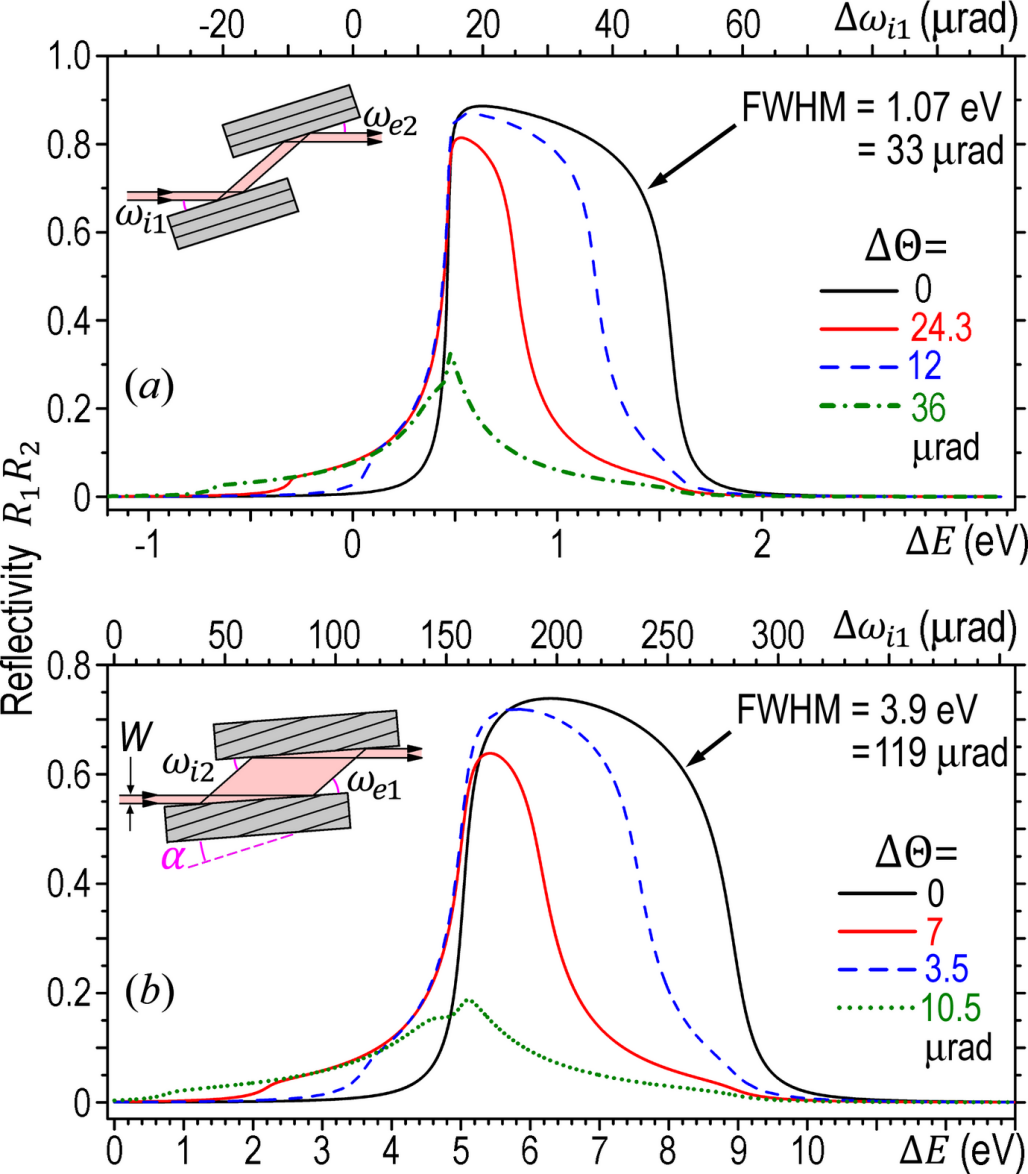
Calculated Darwin curves of the symmetric DCM and the aDCM with different misaligned angles ΔΘ (the angle between the two sets of diffracting planes). ω_i2_ ≡ ω_e1_ + ΔΘ. The Darwin curves of the DCMs are for varying photon energy *E* with the relative orientations of the two crystals fixed. Si 111 reflection for *E* = 8.05 keV. σ-polarization. (*a*) Symmetric DCM. (*b*) aDCM with α = 12.22°.

As shown in Fig. 1 ▸, the two crystal surfaces of an aDCM are both cut by an angle α from the diffracting lattice planes (α = 0 for the standard symmetric DCM). Then the incident angle of the first crystal is 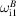 = θ_B_ − α, where θ_B_ is the Bragg angle. A direct consequence of the asymmetric cuts is that the footprint of the X-ray beam on the crystals becomes 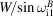, where *W* is the height of the incident beam. For the symmetric DCM, the footprint is 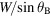. For example, for the Si 111 reflection at *E* = 8.05 keV (θ_B_ = 14.22°), the footprint on the symmetric DCM is 4 mm for *W* = 1 mm. It becomes 28.7 mm and 57.3 mm for α = 12.22° and 13.22° (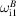 = 2° and 1°), respectively. Consequently, the heat load density can be reduced by about an order of magnitude. Meanwhile, a very beneficial side effect is that the angular acceptance (Darwin width) and the photon energy bandwidth of the grazing-incidence geometry are both increased by a factor of *B* = |*b*|^−1/2^, where *b* = 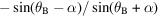 is the asymmetric factor (*B* = 3.6 and 5.1 for α = 12.22° and 13.22°, respectively).

Under the ideal condition ΔΘ = 0, the second reflection of the DCM is exactly the reverse process of the first reflection with ω_i2_ = ω_e1_ and ω_e2_ = ω_i1_, where ω_i*m*_ and ω_e*m*_ are the incidence and exit angle of the *m*th crystal, respectively (*m* = 1, 2). The two reflectivity values are also identical, *R*_2_ = *R*_1_ (only for ΔΘ = 0). The two solid black curves in Fig. 1 ▸ are the calculated curves of the *overall reflectivityR* = *R*_1_*R*_2_ of the DCMs in terms of the relative energy Δ*E* = *E* − *E*_B_, where *E*_B_ is the Bragg energy. These curves are the energy-dependent Darwin curves of the entire DCMs. Compared with Fig. 1 ▸(*a*), the bandwidth of the aDCM in Fig. 1 ▸(*b*) is, indeed, widened by a factor of *B* = 3.6 for α = 12.22° (and ΔΘ = 0).

The angular Darwin curve in terms of Δω_i1_ is the same as the *E*-dependent Darwin curve except that the Δω_i1_ axis is rescaled from the Δ*E* axis by the differential Bragg law Δω_i1_ = 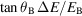. For Fig. 1 ▸, the angular Darwin widths of the aDCM and the symmetric DCM are Ω = 119 and 33 µrad, respectively (not shown), again satisfying the ratio *B* = 3.6. Here note that both the bandwidth broadening and the angular acceptance widening can significantly increase the flux of the aDCM. In particular, if the divergence of the incident beam is larger than the angular Darwin width of the DCM in Fig. 1 ▸(*b*), the total flux is increased from Fig. 1 ▸(*a*) to Fig. 1 ▸(*b*) by nearly a factor of *B*^2^ (instead of *B*). Such a gain may be remarkable and is extremely beneficial for monochromators of laboratory sources or DCMs of high-energy synchrotron sources to achieve very high flux. When the divergence of the incident beam is less than the angular acceptance of the symmetric DCM in Fig. 1 ▸(*a*), only the bandwidth broadening of the aDCM contributes to the flux increase (by a factor of *B*), but the gain can still be substantial for flux-hungry beamlines. Meanwhile, the DCM completely preserves the size, divergence, time structure, coherence and wavefront of the incident beam (Tajiri *et al.*, 2019 ▸).

Note that the angular acceptance of the first and second crystals in Fig. 1 ▸(*b*) are Ω_1_ = *B*Ω_sym_ and Ω_2_ = Ω_sym_/*B*, respectively, where Ω_sym_ is the angular Darwin width of the symmetric reflection in Fig. 1 ▸(*a*). However, the angular acceptance Ω of the entire aDCM is the same as Ω_1_. For α > 0 in Fig. 1 ▸(*b*), the much narrower acceptance of the second crystal (Ω_2_ = 10 µrad) does not affect the wide acceptance of the entire DCM (Ω = 119 µrad) because the first crystal acts as a collimator that reduces the divergence of the incident beam by a factor of *B*^2^ = |*b*|^−1^. Accordingly, the beam received by the second crystal is highly collimated. However, this is only valid for the ideal case where the two crystals are exactly parallel. If they have a misaligned angle ΔΘ, the collimated beam from the first crystal may easily shift out of the narrow angular acceptance range of the second crystal, thus dramatically reducing the efficiency of the DCM.

The Darwin curves of the symmetric DCM for different misaligned angles ΔΘ are shown in Fig. 1 ▸(*a*). The total flux of the DCM is determined by the integrated area below each curve. Compared with the ideal case ΔΘ = 0, the total flux of the red curve is reduced by half when ΔΘ = 24.3 µrad (about two-thirds of the ideal Darwin width Ω_2_). When ΔΘ further increases to 36 µrad (∼Ω_2_), the total flux drops to only 22% of the ideal flux (the dotted-line curve). To achieve high efficiency, therefore, the misaligned angle must be as small as possible. When ΔΘ = 12 µrad (∼Ω_2_/3) in Fig. 1 ▸(*a*), the total flux is about 80% of the ideal flux (the dashed-line curve). We may set this value as the criterion of the DCM, *i.e.* the mis­aligned angle of a DCM must be less than Ω_2_/3 to achieve adequate efficiency.

For the aDCM, Fig. 1 ▸(*b*) shows the three corresponding Darwin curves with reduced efficiency values of 80%, 50% and 22%, respectively, in terms of the ideal efficiency. Compared with Fig. 1 ▸(*a*), the three corresponding misaligned angles are ΔΘ = 3.5 µrad (∼Ω_2_/3), 7 µrad (∼2Ω_2_/3) and 10.5 µrad (∼Ω_2_), respectively. These values are much smaller than those in Fig. 1 ▸(*a*), indicating that the aDCM has more stringent requirement for the misaligned angle than the symmetric DCM. This results from the (much) narrower angular acceptance Ω_2_ of the second crystal in the large-incidence geometry. Here the requirement for the misaligned angle is still ΔΘ 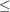

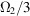
 to achieve an efficiency better than 80% of the ideal efficiency. The criterion of ΔΘ 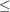

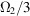
 is approximately valid for any DCMs.

Note that, even if the two crystals are ideally parallel to each other, slight lattice constant difference Δ*d* between the two crystals (caused by different temperatures) can also cause an equivalent misaligned angle ΔΘ = 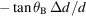. Therefore, channel-cut designs without the relative crystal orientation tweaking capability for compensating temperature-induced ΔΘ should be avoided for high-heat-load DCMs, which is particularly critical for aDCMs.

Thus, we have revealed that aDCMs have stricter requirements for alignment and stability of the relatively crystal orientation than symmetric DCMs. Nevertheless, these requirements are usually on the µrad level that are achievable in practice. Therefore, aDCMs are practical monochromator schemes that can significantly reduce heat load density and thermal gradient, particular for fourth-generation synchrotrons, together with the benefits of large bandwidth and angular acceptance and complete preservation of the beam size, divergence and coherence.

## 3. Properties of iDCMs

### 3.1. Rigorous dynamical theory calculations of inclined diffraction

**Figure 2:**
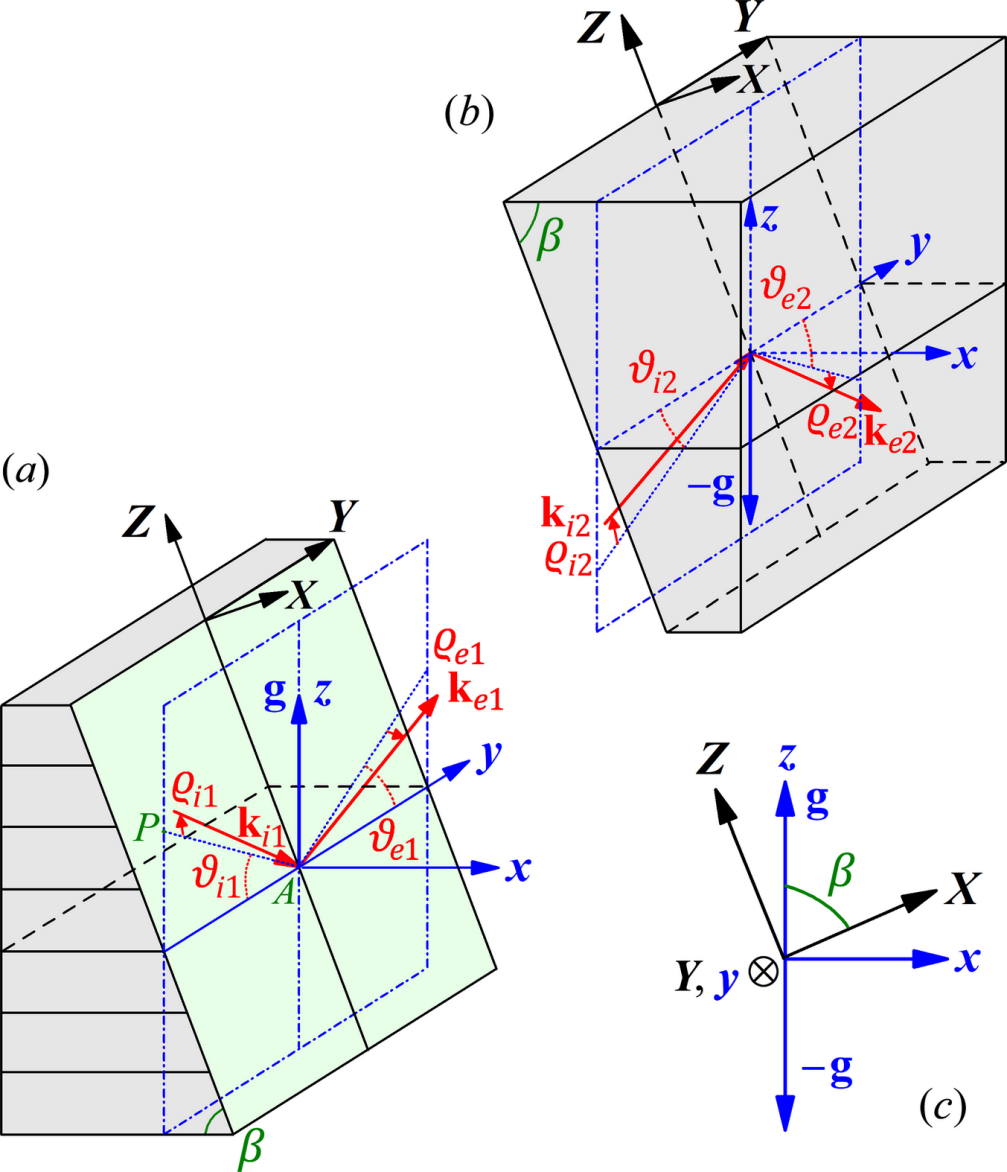
Schematic of X-ray diffraction of the iDCM (β = 0 corresponds to a conventional symmetric DCM). (*a*) The first crystal of the iDCM. *PA* is the projection of **k**_i1_ onto the *yz* plane. ϑ_i1_ is the angle between *PA* and the *y*-axis. 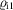 is the angle between **k**_i1_ and *PA*. 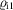  ≥ 0 if *k*_i1*z*_ ≥ 0, and 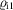 < 0 if *k*_i1*z*_ < 0. The angles of other wavevectors have similar definitions. (*b*) The second crystal with the same β. **k**_i2_ = **k**_e1_. Changing energies of the DCM is by the ϑ-rotation of the two crystals together around the *x*-axis. (*c*) Relationship between the *xyz* and *XYZ* coordinate systems.

In addition to the above *coplanar* aDCMs, iDCMs have been proposed as another type of grazing-incidence monochromator for reducing heat load density (Khounsary, 1992 ▸; Hrdý, 1992 ▸). Fig. 2 ▸(*a*) shows the symmetric inclined diffraction geometry, where the *xy* plane is parallel to the diffracting lattice planes, and the *yz* plane is the *plane of diffraction* determined by the diffraction vector **g** (parallel to the *z*-axis) and the principal incident wavevector **k**_i1_. The crystal surface is the *YZ* plane inclined from the *yz* plane by 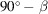 around the −*y* axis, where β is the inclined angle of the crystal surface from the *xy* plane. Here, since the surface normal (the *X* axis) is not in the *yz* plane of diffraction, it is a *non-coplanar diffraction configuration*. Under the Bragg condition, we have ϑ_i1_ ≃ 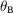 (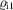 ≃ 0), but the actual *glancing angle* (not marked) is the angle between **k**_i1_ and the *YZ* plane,
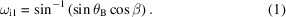
Obviously, ω_i1_ → 0 for any θ_B_ when 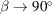. In experiments, the θ-scan is to rotate the crystal around the *x*-axis. For convenience, we will consider ϑ_i1_ and ϑ_e1_ as the incidence and exit angles with respect to the *xy* plane (rather than the crystal surface), respectively, in the following. Here we have ϑ_e1_ ≃ ϑ_i1_ such that the diffraction configuration is still symmetric with respect to the *xz* plane. Unlike asymmetric diffraction, the symmetric inclined diffraction geometry in Fig. 2 ▸ has almost the same energy tuning range as regular symmetric diffraction (β = 0) because here ϑ_i1_ can still cover the entire 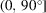 range.

Dynamical theory calculations of inclined diffraction have been a difficult task in the literature. Afanas’ev & Melkonyan (1983 ▸) developed an approximate method that can treat the diffraction configuration in Fig. 2 ▸(*a*), but it is valid only for 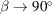. Later, Huang *et al.* (2013 ▸) developed the fully vectorial *Fourier coupled-wave diffraction theory* (FCWDT) that can rigorously treat general *N*-beam diffraction from periodic structures (including optical diffraction from photonic crystals) in 3D space. This method is for solving the eigenmodes of the coupled Fourier components of the electric fields (**E**) and the magnetic fields (**H**), and the strengths of these fields are determined by the boundary conditions of the **E** and **H** fields. The FCWDT can calculate any coplanar or non-coplanar X-ray diffraction involving two or more beams without approximation. Here we adopt this method.

**Figure 3:**
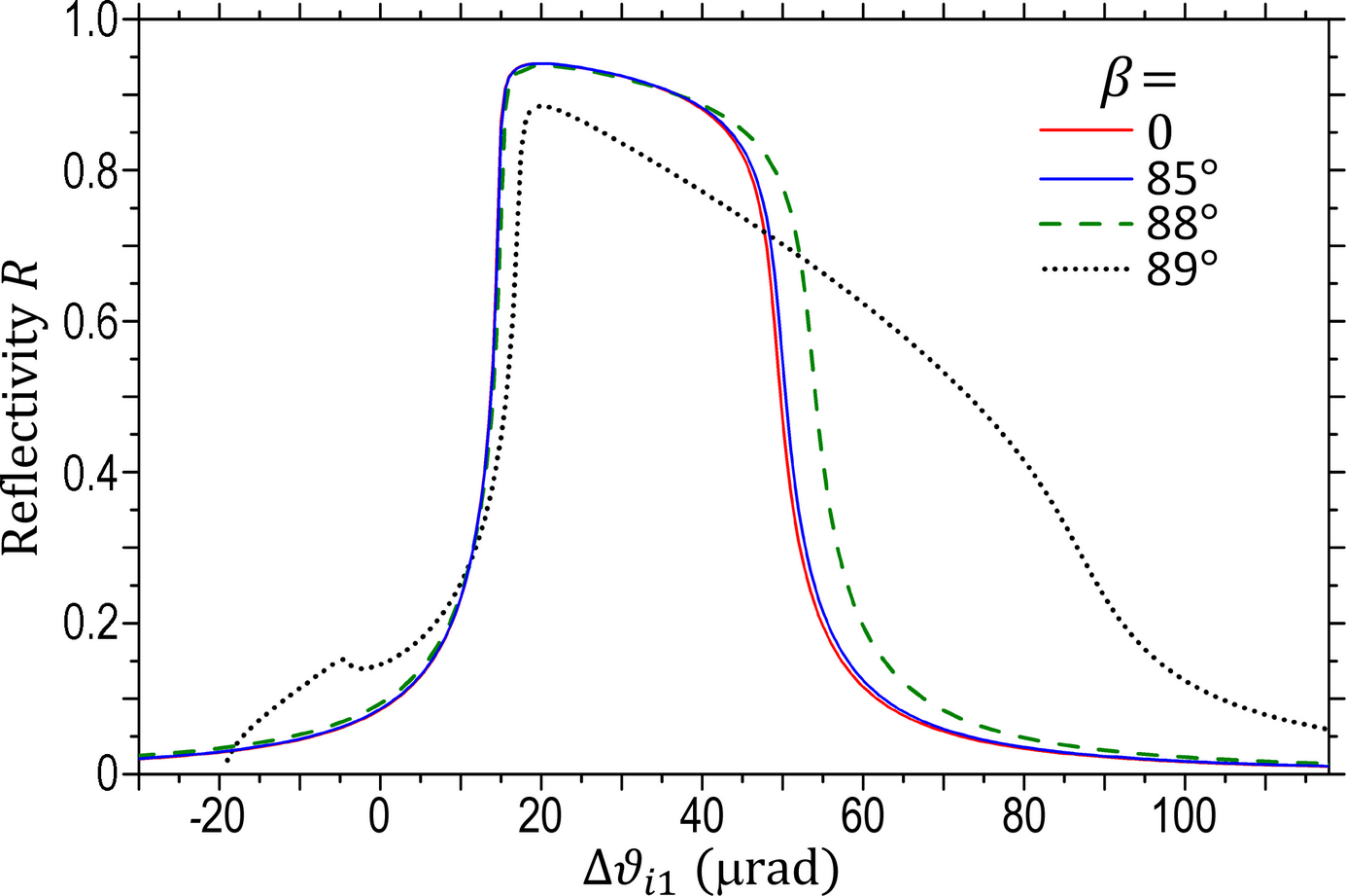
Darwin curves of single-bounce Si 111 inclined diffraction calculated by FCWDT based on the geometry of Fig. 2 ▸(*a*) (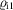

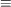
 0). *E* = 8.05 keV. σ-polarization.

Fig. 3 ▸ shows the calculated Darwin curves of the Si 111 reflection for different inclined angles β. The Darwin curve for β = 0 precisely coincides with that calculated by the conventional dynamical theory, which directly verifies the reliability and accuracy of the FCWDT. Our calculations show that all the Darwin curves for 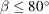 have no noticeable difference from the β = 0 curve in Fig. 3 ▸. Only when 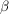

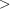

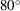
 does the Darwin curve start to show deviations. For example, the blue curve for β = 85° in Fig. 3 ▸ shows a slight difference on the right shoulder. The difference becomes evident when β increases to 88°. The Darwin curve for β = 89° differs further and becomes much wider, corresponding to wider angular acceptance and a proportionally larger bandwidth (not shown).

Note that the glancing angles of the incident beam for β = 80°, 85°, 88° and 89° are ω_i1_ = 2.44°, 1.23°, 0.49° and 0.25°, respectively, according to equation (1). Apparently, the deviation of the Darwin curves for 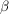

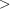

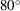
 are caused by X-ray specular reflection from the crystal surface when ω_i1_ is very small (typically 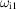

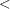
 1°). For 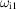

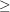
 1°, the specular reflection is negligible, and the inclined crystal performs the same as the corresponding regular coplanar geometry in terms of reflectivity, which can be verified by FWCDT calculations.

As indicated by Fig. 3 ▸, the extremely inclined geometry (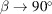, corresponding to extremely grazing-incidence diffraction) still preserves high reflectivity. Practically this geometry is very useful to achieve very large beam footprint magnification (for example, the magnification for β = 89° is 229) (Khounsary, 1992 ▸). Meanwhile, it has the extra benefit of a larger bandwidth and angular acceptance, which is valuable for flux-hungry beamlines. The extremely grazing-incidence geometry requires long super-smooth crystal surfaces (similar to X-ray mirrors), but this is feasible based on modern crystal polishing techniques.

### 3.2. The ‘rho-kick’ effect

**Figure 4:**
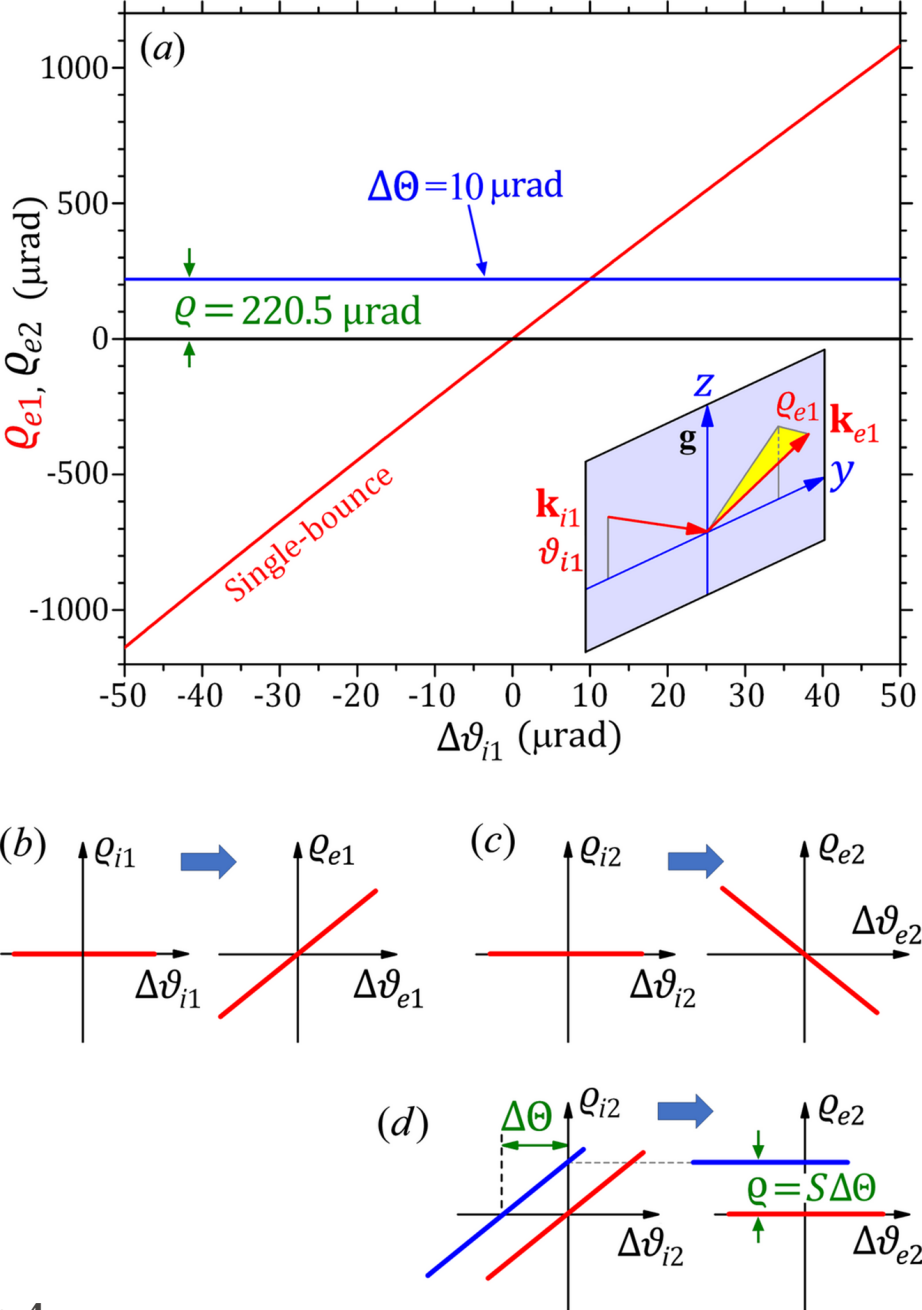
The ‘rho-kick’ effect of iDCMs. (*a*) The red line is the rho-kick curve 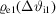 for single-bounce Si 111 inclined diffraction. The two horizontal lines are the rho-kick curves 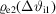 of the entire two-bounce DCM with ΔΘ = 0 and 10 µrad, respectively. Calculated by the FCWDT based on Fig. 2 ▸(*a*). 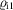

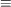
 0. β = 85°. *E* = 8.05 keV. The inset schematically shows the rho-kick angle 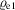. The *yz* plane is the same as that in Fig. 2 ▸(*a*). (*b*, *c*) The rho-kick functions of the two reflections with opposite slopes (for 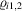

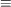
 0). (*d*) The rho-kick functions of the second reflection of the DCM with 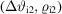 = 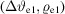 (red lines) and 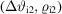 = 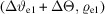 (blue lines).

Inclined diffraction has the following subtle difference even for 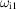

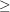
 1°, the ‘rho-kick’ effect. This effect was revealed by Lee *et al.* (1996 ▸), and can be illustrated as follows. Even if the incident wavector **k**_i1_ in Fig. 2 ▸(*a*) strictly lies within the *yz* plane (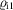 = 0), the diffracted wavevector **k**_e1_ can have an out-of-plane component with respect to the *yz* plane, *i.e.*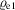 ≠ 0, as schematically shown in the inset of Fig. 4 ▸(*a*). Moreover, 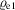 may vary dramatically with the incidence angle ϑ_i1_. In Fig. 2 ▸, this effect can be computed by the following angular tracing.

We start from the wavevector **k**_i1_ in Fig. 2 ▸(*a*) with an incidence angle ϑ_i1_ and, for generality, we allow **k**_i1_ to have a small out-of-plane angle 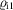. In the *xyz* coordinate system, **k**_i1_ can be written as
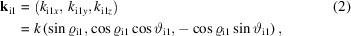
where *k* = 1/λ (λ the incident wavelength). In the *XYZ* coordinate system, **k**_i1_ is transformed from equation (2) into
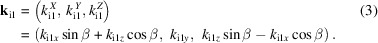
The key principle for determining the diffracted wavevector **k**_e1_ is the *conservation of the tangential wavevectors* with respect to the crystal surface (Huang *et al.*, 2013 ▸; Huang *et al.*, 2012 ▸),
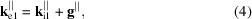
where ‘
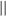
’ means the projection of the vector onto the crystal surface (the *YZ* plane). Based on this principle, the diffracted wavevector in the *XYZ* system is 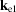 = 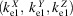 with 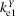 = 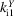,
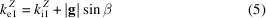
and
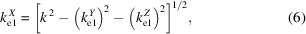
where |**g**| = 1/*d* (*d* is the spacing of the diffracting lattice planes). Afterwards, we can transform **k**_e1_ back to the *xyz* system,
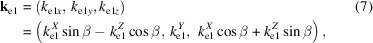
which gives the two angles 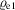 = 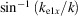 and ϑ_e1_ = 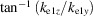.

Now we let the second crystal of the iDCM in Fig. 2 ▸(*b*) receive this diffracted wave. When the two crystals are exactly parallel to each other, ϑ_i2_ = ϑ_e1_ and 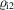 = 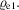 In the *xyz* coordinate system, the incident wavevector of the second crystal is
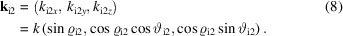
In the *XYZ* system, it becomes
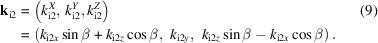
Based on equation (4), we can obtain the components of **k**_e2_ = 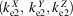 in the *XYZ* system as 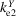 = 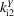,
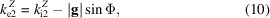
and
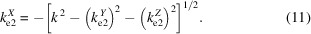
Here, note that the diffraction vector is −**g** in Fig. 2 ▸(*b*), which results in the ‘−’ sign in equation (10). Transforming **k**_e2_ from the *XYZ* system to the *xyz* system gives
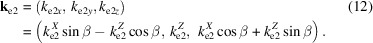
Then the two angles of the exit wave are 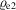 = 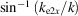 and ϑ_e2_ = 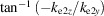. The angular tracing is automatically implemented in the FCWDT. Here we have explicitly derived the equations, which can be used for rigorous ray tracing of iDCMs (Blasdell *et al.*, 1994 ▸).

The red-line curve in Fig. 4 ▸(*a*) is the calculated rho-kick angle 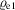 as a function of the relative incidence angle Δϑ_i1_ for highly inclined Si 111 single reflection (with 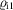 ≡ 0). The curve is nearly a straight line and its slope is *S* = 22.1 in the vicinity of the Bragg angle. For a fixed inclined angle β, the slope *S* remains nearly constant for different energies (*E*). However, *S* quickly decreases with decreasing β (*e.g.S* = 9.1 and 5.5 for β = 78° and 70.5°, respectively, not shown). These results agree well with those given by Lee *et al.* (1996 ▸).

The large value *S* = 22.1 of the single-bounce curve in Fig. 4 ▸(*a*) indicates that the rho-kick angle changes significantly with slightly varying Δϑ_i1_. As shown in Fig. 3 ▸, the Darwin width for the Si 111 reflection is 40.2 µrad at 8.05 keV. Even if the incident beam has no horizontal divergence (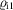 ≡ 0), this vertical angular width will cause a highly amplified horizontal divergence of 888 µrad for the exit beam, as shown schematically in Fig. 4 ▸(*b*). This is the rho-kick effect of inclined diffraction that may severely change the shape, divergence and brightness of the X-ray beam, together with other issues including virtual source broadening, focusing, coherence *etc*.

Fortunately, for a double-bounce iDCM, the second reflection is the reversed process of the first one. For an incident beam with 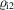 ≡ 0, the second reflection will produce a rho-kick line with slope −*S*, as shown in Fig. 4 ▸(*c*). The rho-kick functions of the two crystals can be written as
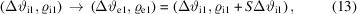

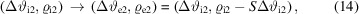
respectively, where Δϑ_*m*_ = ϑ_*m*_ − θ_B_ for *m* = i1, e1, i2, e2. Here we have Δϑ_e1_ ≃ Δϑ_i1_ and Δϑ_e2_ ≃ Δϑ_i2_. Under ideal conditions, the exit beam of the first reflection and the incident beam of the second reflection have identical directions, 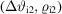 = 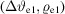 = 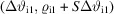. Substituting this relation into equation (14) gives 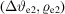 = 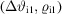, *i.e.* the exit beam of the second reflection preserves the exact direction of the incident beam of the first reflection. The red lines in Fig. 4 ▸(*d*) schematically show this process for 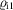 ≡ 0, which is strictly verified by the rigorously calculated black line 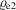 ≡ 0 in Fig. 4 ▸(*a*). Therefore, a perfect iDCM has no rho-kick distortion, and it precisely preserves the direction, shape, brightness and wavefront of the incident beam.

However, if the second crystal has a misaligned angle ΔΘ relative to the first crystal (*i.e.* the second crystal in Fig. 2 ▸ is rotated around the *x*-axis by ΔΘ), the incidence direction of the second crystal becomes 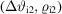 = 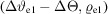 = 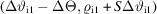. In Fig. 4 ▸(*d*), this corresponds to a shift of the incident beam profile of the second crystal to the left. Substituting this relation into equation (14) gives
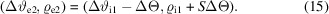
With respect to the incidence direction 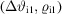, here the shift of the 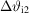 angle by ΔΘ causes a constant rho-kick angle
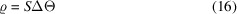
for the exit beam of the DCM, which is schematically shown in the Fig. 4 ▸(*d*). The blue line in Fig. 4 ▸(*a*) is the rigorously calculated 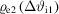 curve for ΔΘ = 10 µrad, which is a horizontal line with 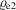 ≡ 220.5 µrad, very close to the value 221 µrad given by equation (16). Therefore, a relative ΔΘ angle between the two crystals of an iDCM (which may be caused by crystal misalignment or a uniform lattice constant difference) can lead to a constant rho-kick angle for the exit beam, which does not affect the beam shape, divergence or brightness.

In the above, we have discussed the diffraction process of a single wavelength. For a practical incident beam with a finite bandwidth, most of the spectral components do not satisfy the exact Bragg condition (*i.e.*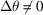), and each component has a different rho-kick angle. For a single inclined diffraction configuration, this dispersion effect can also significantly elongate the virtual source size. However, for an ideal iDCM, the second reflection always exactly cancels out the rho-kick angle for each spectral component because the above angular tracing principle applies to each component. It is also independent of the Bragg angle, although the deviation of each component from the Bragg angle and Bragg wavelength affects the diffraction intensity.

### 3.3. Tolerance to misalignments

**Figure 5:**
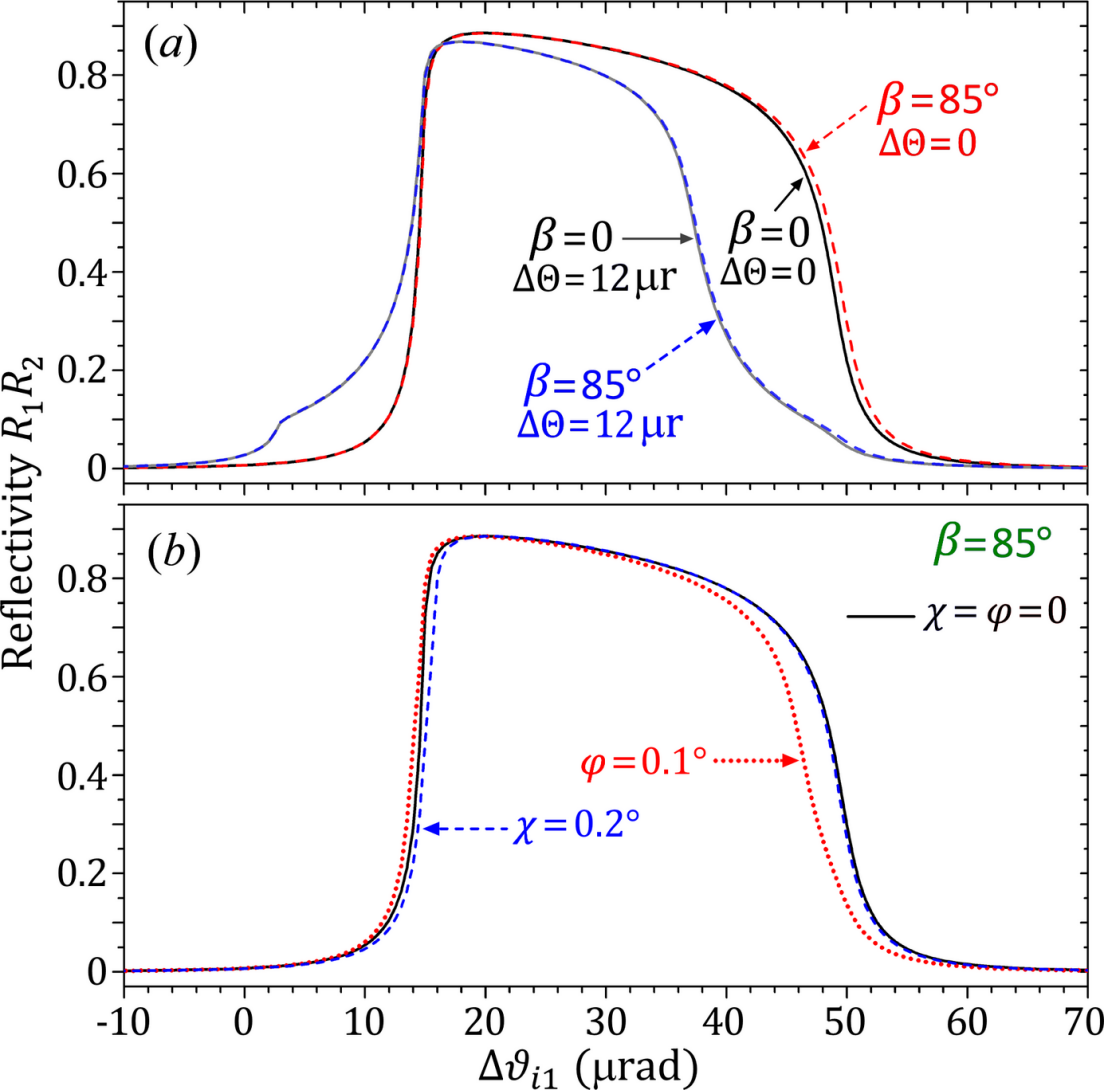
(*a*) Similarities between the Darwin curves of the iDCM and the normal DCM with and without misalignment ΔΘ. Si 111 reflection. *E* = 8.05 keV. σ-polarization. The two Darwin curves for β = 0 are the same as the corresponding curves in Fig. 1 ▸(*a*). (*b*) Tolerance of the iDCM Darwin curve to the relative misalignment angles χ and φ.

The Darwin curves calculated by the rigorous FCWDT for the iDCM with β = 85° are shown in Fig. 5 ▸ in comparison with those of the normal DCM (β = 0). Similar to Fig. 3 ▸, the double-reflection Darwin curves of the iDCM are very close to those of the normal DCM. To a good approximation, therefore, one again can safely use the simple conventional dynamical theory to estimate the reflectivity and other properties of an iDCM if the glancing angle satisfies ω_i1_ ≥ 1°. These two types of DCMs have almost the same Darwin width, angular acceptance and bandwidth, which are independent of β unless β is very close to 90°. This is also true when the two crystals are slightly misaligned by an angle ΔΘ, as shown by the two curves for ΔΘ = 12 µrad. As mentioned above, at this angle the integrated flux (efficiency) is reduced to 80% of the ideal value. Therefore, these two types of DCMs have the same sensitivity to ΔΘ. The difference is that the iDCM produces a uniform rho-kick angle 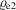 = *S*ΔΘ, as demonstrated above.

X-ray diffraction from iDCMs is a 3D non-coplanar configuration, which may also be affected by the relative misaligned angles around other axes. The blue dash line in Fig. 5 ▸(*b*) is the Darwin curve of the iDCM when the second crystal in Fig. 2 ▸(*b*) is rotated around the *y*-axis by χ = 0.2° (usually called the tilt angle). Apparently, the difference between the two Darwin curves for χ = 0 and 0.2° is negligible, indicating that iDCMs are quite insensitive to the relative tilt misalignment. The red dotted line in Fig. 5 ▸(*b*) is the Darwin curve when the second crystal in Fig. 2 ▸(*b*) is rotated around the *z*-axis by an angle φ = 0.1°. This curve shows some noticeable difference from the ideal Darwin curve on the right shoulder, indicating that the iDCM is moderately sensitive to the φ misalignment. The underlying mechanism is that the φ angle directly affects the glancing angle ω_i2_ of the incident beam on the second crystal, which can be understood from Fig. 2 ▸. The misaligned angle φ = 0.1° also causes the rho-kick curve 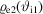 to have a small slope *S* = 1.9, and the slope caused by χ = 0.2° is *S* = 0.85 (not shown). These two slope values are much smaller than the value *S* = 22.1 caused by the single reflection in Fig. 4 ▸(*a*). Overall, iDCMs are not very sensitive to either χ or φ misalignment, but are extremely sensitive to the ΔΘ misaligned angle.

## 4. Discussion of crystal strains

We have demonstrated that iDCMs have almost the same properties as normal symmetric DCMs, and these properties are nearly independent of the inclined angle β (except for the extreme cases with 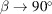 or ω_i1_ → 0). Thus, one can freely choose the desired β angle to adjust the beam footprint (power density) on the crystal without affecting other properties of the iDCMs. The only exception is that iDCMs are much more sensitive to *inhomogeneous strains* (lattice distortions) possibly induced during crystal fabrication and mounting, or by thermal gradients during operations. Inhomogeneous strains, even on the µrad level, may noticeably reduce the efficiency of iDCMs, which is also true for aDCMs. In particular, inhomogeneous strains of iDCMs can cause varying misaligned angles ΔΘ(**r**) across the beam footprint on the crystal, thus producing non-uniform rho-kick angles 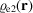 = *S*ΔΘ(**r**) for the exit beam.

**Figure 6:**
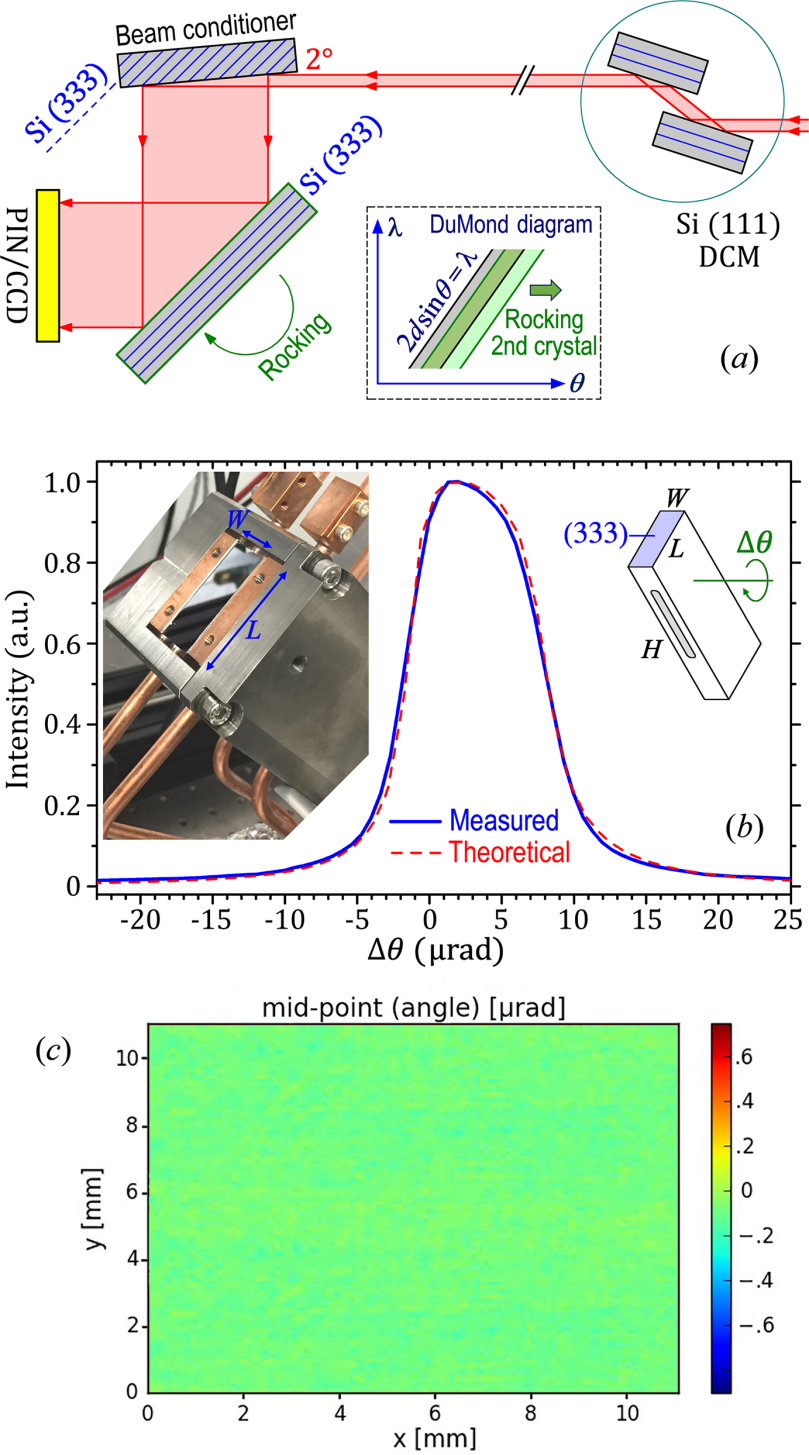
(*a*) Schematic of the double-crystal rocking curve imaging technique. (*b*) Measured and calculated double-crystal rocking curve of a cryogenic Si (111) monochromator (left inset). Crystal dimension 40 mm (L) × 15 mm (W) × 95 mm (H) (right inset). The X-ray footprint on the crystal is about 35 mm × 12 mm during measurements. (*c*) The distribution map of midpoints of local rocking curves extracted from rocking curve images recorded by a CCD camera with 4096 × 4096 pixels (pixel size 2.6 µm). Si 333 reflection. *E* = 8.05 keV. σ-polarization.

In the earlier days it was estimated that the fabrication/mounting-induced strains in DCMs could be on the level of ∼6 µrad, which was the major source of strains worse than the thermal strains (Lee *et al.*, 1996 ▸). Fortunately, after more than two decades of improvements, nowadays DCMs made of high-quality float-zone-grown silicon crystals can be nearly free of fabrication/mounting-induced strains, which may be verified by the double-crystal rocking curve imaging method illustrated in Fig. 6 ▸(*a*). This is the most accurate method for detecting and mapping crystal strains. Here the first crystal is the beam conditioner with an incidence angle ∼2°, which also acts as the beam expander/collimator. The second crystal must have the same reflection (Bragg angle). Then the diffraction bands of the two crystals in the DuMond diagram are parallel, and their overlap during crystal rocking is the same for any wavelength λ. Thus, the measured rocking curve of the second crystal is almost independent of the bandwidth and divergence of the incident beam, which ensures the reliability of this method regardless of experimental conditions. Moreover, the measured rocking curve width of a perfect crystal is very close to the theoretical Darwin width that can be only a few µrad. Consequently, the strain sensitivity can reach the sub-µrad level. For example, the theoretical Darwin width of symmetric the Si 333 reflection is 9.63 µrad for *E* = 8.05 keV, and the width of the convoluted double-crystal rocking curve is only slightly broader at 9.90 µrad. Another major advantage is that this method can map strains over large areas (up to 80 mm × 80 mm in our experiments), which is critical for characterization of aDCMs and iDCMs that requires large crystal surfaces to be strain-free.

As an example, Fig. 6 ▸(*b*) shows the measured double-crystal rocking curve of a symmetric Si (111) monochromator installed on a cryogenic cooling fixture with indium foil thermal interfaces. The measurement was carried out at beamline 1-BM of the Advanced Photon Source (APS) at room temperature with negligible heat load. Here the 333 reflection rocking curve width of 9.9 µrad over the entire footprint of 35 mm × 12 mm perfectly matches the theoretical convoluted rocking curve width. Moreover, the shape of the measured curve is also in good agreement with the theoretical shape. Note that the asymmetric shape of the rocking curve results from the absorption-induced asymmetry of the Darwin curve. This asymmetric profile is precisely repeatable in our experiments, which indicates the high accuracy of the double-crystal diffraction method. Here the strain sensitivity of this method is better than 0.5 µrad. At this precision level, Fig. 6 ▸(*b*) shows no noticeable strains over the entire footprint, indicating that the fabrication/mounting-induced strains of the crystal are negligible (<0.5 µrad). Currently these results can be routinely achieved for silicon monochromators made at the APS.

Note that the integrated rocking curve in Fig. 6 ▸(*b*) only shows that the crystal has no noticeable ‘global’ strain. In our rocking curve imaging experiments, we used a charge-coupled device (CCD) to record a series of images of the diffracted beam during the rocking curve scan. Thus, each pixel of the CCD recorded a *local* rocking curve. Numerical processing of all the local rocking curves then gives detailed maps revealing the local peak reflectivity, local rocking curve widths, midpoints of the local rocking curves, *etc*. From these maps local information of lattice strains (and also defects) can be clearly extracted (Stoupin *et al.*, 2019 ▸). For example, the map in Fig. 6 ▸(*c*) is extracted from the CCD data revealing the distribution of the midpoints of the local rocking curves, which indeed shows that the crystal has no noticeable local strains within the sub-µrad precision.

Another fact that confirms the feasibility of achieving strain-free silicon monochromators is the successful implementation of ultrahigh-resolution monochromators with meV or even sub-meV energy resolution (Yabashi *et al.*, 2001 ▸; Toellner *et al.*, 2006 ▸; Toellner *et al.*, 2011 ▸). To achieve such resolution, the crystal strains must be below 10^−7^ (0.1 µrad) over the entire footprints. Here, note that the X-ray footprints of these monochromators are also quite large since they use extremely asymmetric diffraction geometry. Nevertheless, these monochromators are not under direct high heat load but instead work downstream of high-heat-load DCMs. Their successful implementation does not resolve the concern of the thermal strains induced by high heat load, which is the other major type of strain.

However, two factors may help mitigate thermally induced strains in iDCMs. First, high-heat-load iDCMs for fourth-generation synchrotrons are expected to be cryogenically cooled, preferably around 123 K when the thermal expansion coefficient of silicon goes to zero (Toellner *et al.*, 2006 ▸). Around this temperature, the thermal strains (lattice constant gradients) induced by temperature gradients are minimized.

The second factor is the iDCM itself, which is designed to reduce the heat load density and gradient by elongating the footprints on the crystals. With the footprint elongated to a few centimetres on the crystal, the thermal gradient along the elongation direction will be minimum except for the areas near the two ends (of which the X-ray diffraction contribution is small). Consequently, the local deviation of the Bragg angle, ΔΘ, which is mainly induced by the thermal gradient along the elongation direction, is minimized. For a small incident beam, the spread footprint has a line shape on the crystal surface, and the thermal gradient along the direction perpendicular to the line may still be notable. However, for relatively small Bragg angles, the inhomogeneous strains along this direction mainly contribute to deviation of the tilt angle χ, which has little effect on the performance of the iDCM or the rho-kick angle, as shown in Fig. 5 ▸(*b*). Note that these discussions are also applicable to aDCMs.

Overall, combined with cryogenic cooling, we believe that high-heat-load iDCMs with large beam footprints can achieve low thermal strains of the order of 1–2 µrad, which is supported by the fact that the heat load density can be reduced by about an order of magnitude from symmetric DCMs to iDCMs. But this claim requires future experimental verification.

Similar to the fact that an iDCM has an angular amplification rate of *S*, *i.e.* the rho-kick slope (along the horizontal direction for vertical diffraction), for the relative orientation variation between the two crystals, an aDCM also has an angular amplification rate of |*b*|^−1^ (along the vertical direction) for the relative orientation change. The underlying mechanism is that, when the first crystal of the aDCM has a small rotation δθ, the incidence angle is changed by δθ. Due to the collimation effect of the grazing incidence geometry, the exit angle is changed by δθ|*b*| with respect to the first crystal surface. The total rotation of the first exit beam is then δθ(|*b*| + 1) relative to the (fixed) second crystal. As the reversed process of the first crystal, the second crystal has an angular amplification rate of |*b*|^−1^ that amplifies this angular variation to be δθ(|*b*| + 1)/|*b*| ≃ δθ/|*b*|. This mechanism has been verified by our detailed *SHADOW* simulation (Sanchez del Rio *et al.*, 2011 ▸). When non-uniform strains or thermal bumps are present, these two angular amplification phenomena may have noticeable influence on beam pointing stability in addition to its influence on the virtual source, the beam brightness, *etc*. Nevertheless, all these effects should be minimum for aDCMs and iDCMs because the thermal bumps have been significantly reduced by the large beam footprints on the crystals. In our future work, we plan to use finite-element analyses to quantify the thermal bumps on aDCMs and iDCM and study their influence on beam pointing. Eventually we will experimentally test the aDCM and iDCM at fourth-generation synchrotron beamlines.

## 5. Summary

We have provided a relatively comprehensive description of aDCMs and iDCMs based on rigorous dynamical theory calculations. The common feature of aDCMs and iDCMs is that they have larger beam footprints on the crystals, corresponding to much lower heat load density and gradient (by about an order of magnitude) than the conventional DCMs. However, both types of DCMs require much lower inhomogeneous crystal strains than the normal DCMs. The aDCM have large bandwidth (up to ∼10 eV) and angular acceptance determined by the first crystal in the grazing-incidence geometry, but the narrow angular acceptance of the second crystal requires high precision of alignment (and stability) of the relative orientations between the two crystals to achieve adequate efficiency. The total misalignment and inhomogeneous strains must be controlled typically within 4 µrad (<Ω_2_/3). For iDCMs, we used the rigorous FCWDT calculations to precisely prove that they have almost the same properties as conventional symmetric DCMs, including the efficiency, angular acceptance, bandwidth, energy tuning range and sensitivity to crystal misalignment. The exception is that extremely inclined iDCMs (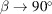) have wider bandwidths and angular acceptance. Inclined diffraction has the rho-kick effect, in which the vertical incident beam divergence can produce highly amplified divergence of the exit beam along the horizontal direction. But this effect can be perfectly cancelled by the two-bounce iDCM as the second reflection cancels out the rho-kick angle of the first reflection. This cancellation is largely valid even if the two crystals have misalignment. Under favourable conditions, therefore, iDCMs can well preserve the beam shape, divergence, brightness and coherence. The only challenge is that iDCMs are very sensitive to inhomogeneous strains that cause non-uniform rho-kick angles to degrade the beam brightness. We demonstrated that fabrication/mounting-induced crystal strains can be controlled to the level <0.5 µrad over large areas. We expect that, by combining cryogenic cooling, the other major type of strains, the thermally induced strains, can also be controlled to the 1 µrad level so that the inhomogeneous rho-kick angles are within ∼20 µrad (for β ≃ 85°). Note that this requirement is relaxed quickly for decreasing inclined angles β (corresponding to decreasing *S*). In our future work, we plan to experimentally test the iDCM prototypes and to study the thermal strains.

In addition, the rho-kick effect of inclined diffraction can be used as an angular amplification technique (with the amplification rate >20) for many high-resolution X-ray diagnostic applications, including accurate measurements of X-ray beam divergence, crystal strains and angular stability of optical components. The FCWDT method demonstrated above can be used for designing iDCMs and other inclined diffraction optics (Hrdý *et al.*, 2011 ▸; Oberta *et al.*, 2012 ▸; Smither *et al.*, 2012 ▸). It is also capable of computing any non-coplanar two- or multiple-beam X-ray diffraction in 3D space for arbitrary forms of polarization (including elliptical polarization and mixed linear polarization), which is a valuable supplement to the classical dynamical theory.
